# PAX3 Regulatory Signatures and Gene Targets in Melanoma Cells

**DOI:** 10.3390/genes16050577

**Published:** 2025-05-14

**Authors:** Stephen P. G. Moore, Shripushkar Ganesh Krishnan, Rutu Jaswanth Kothari, Noah B. Prince, Colin Kenny, Chao Zhang, Deborah Lang

**Affiliations:** 1 Department of Dermatology, Boston University, Boston, MA 02118, USA; spgmoore@bu.edu (S.P.G.M.); pushkar@bu.edu (S.G.K.); rutu@bu.edu (R.J.K.); nbprince@bu.edu (N.B.P.); 2 Bioinformatics Program, Boston University, Boston, MA 02118, USA; 3 Department of Surgery, University of Iowa, Iowa City, IA 52242, USA; colin-kenny@uiowa.edu; 4 Department of Medicine, Boston University, Boston, MA 02118, USA

**Keywords:** PAX3, melanoma, transcription factor, PAX proteins, transcriptomics, gene regulation

## Abstract

Background/Objectives: PAX3 is a transcription factor that drives melanoma progression by promoting cell growth, migration, and survival, while inhibiting cellular terminal differentiation. However, known PAX3 target genes are limited and cannot fully explain the wide impact of PAX3 function. The PAX3 protein can regulate DNA through two separate binding domains, the Paired Domain (PD) and Homeodomain (HD), which bind different DNA motifs. It is not clear if these two domains bind and work together to regulate genes and if they promote all or only a subset of downstream cellular events. Methods: PAX3 direct downstream targets were identified using Cleavage Under Targets & Release Using Nuclease (CUT&RUN) assays in SK-MEL-5 melanoma cells. PAX3-binding genomic regions were identified through MACS2 peak calling, and peaks were categorized based on the presence of PD and/or HD binding sites (or neither) through HOMER motif analysis. The peaks were further characterized as Active, Primed, Poised, Repressed, or Closed based on ATAC-seq data and CUT&RUN for histone Post-Translational Modifications H3K4me1, H3K4me3, H3K27me3, and H3K27Ac. Results: This analysis revealed that most of the PAX3 binding sites in the SK-MEL-5 cell line were primarily through the PD and connected to Active genes. Surprisingly, PAX3 does not commonly act as a repressor in SK-MEL-5 cells. Pathway analysis identified genes involved with transcription, RNA modification, and cell growth. Peaks located in distal enhancer elements were connected to genes involved in neuronal growth, function, and signaling. Conclusions: Our results reveal novel PAX3 regulatory regions and putative genes in a melanoma cell line, with a predominance of PAX3 PD binding on active sites.

## 1. Introduction

PAX3 is a transcription factor expressed in the embryonic neural crest, somites, and central nervous system (CNS) [1]. Homozygous deletion of PAX3 leads to defects in all these populations in mice. PAX3 is linked to driving lineage specificity genes as the somites and neural crest differentiate and mature, with examples including *MyoD* in the somite derived-muscle precursors and *MITF* in neural crest-derived melanocytes [2,3,4,5,6]. PAX3 is required for normal melanocyte development, growth, and migration. Hair hypopigmentation and hearing loss occurs in mice or Waardenburg Syndrome patients with hemizygous *PAX3/pax3* gene loss or mutation due to missing or defective melanocytes [7]. On the other hand, PAX3 expression is maintained in melanoma, in early lesions, primary tumors and metastasis [8,9,10,11]. The overexpression of PAX3 in melanoma is not purely an artifact of a prior initiating event of downstream effector genes, since the loss of PAX3 is catastrophic to melanoma viability regardless of expression of these downstream factors [8,9,10,11].

The main function of PAX3 is as a transcription factor, but most downstream targets are not known. While PAX3 target genes have been identified using a candidate approach, non-biased cistromic screens have been limited overall and are nonexistent for melanocyte or melanoma cells. The few studies utilizing either ChIP-seq, CUT&RUN, or CUT&Tag were mostly focused on the role of PAX3 in muscle cells or embryonic stem cells induced toward a muscle lineage [12,13,14]. Genomic PAX3 binding within *xenopus* neural crest was also examined in a recent report [15]. In cancer cells, current studies have focused on Fusion Positive Rhabdomyosarcoma (FP-RMS) containing the PAX3-FOXO1 translocation product [16,17,18,19,20,21,22,23,24,25]. While these studies are informative, it is not clear if the identified enhancers and genes are normal PAX3 targets or unique to the translocation protein, where the C-terminal tail and transactivation domain is replaced with significant FOXO1 regulatory regions. While several genes have been identified as putative PAX3 targets, only a handful have been verified as direct PAX3 downstream targets with linked enhancer regulatory elements. In neural crest, melanocyte, and melanoma cells, PAX3 was found to regulate *MITF*, *NGN2*, *RET*, *SOSTDC1*, *MSX2*, *DCT*, and *TYRP1* genes [5,6,26,27,28,29,30,31,32], as well as genes expressed ubiquitously or widely (and including melanocytes), such as *BCL-XL*, *CXCR4*, *FGFR4*, *HES1*, *MET*, *NF1*, *TGFb2*, and *WNT1* [11,30,33,34,35,36,37,38,39,40,41,42]. While PAX3 is important in both melanocytes and melanoma, gene signature studies have not been performed on a whole genome scale. Clearly, the PAX3-dependent influence on melanoma maintenance and progression is much wider than the few known downstream genes already identified.

In this current study, we focused on the SK-MEL-5 cell line as a model for melanoma, since these cells display common cellular morphology for this cell type and retain a BRAF^V600E^ mutation that is found in about half of melanoma cases [43,44]. The majority of PAX3 peaks contained PD binding sites, were affiliated with active genes, and were within 5 Kb of a Transcriptional Start Site (TSS). Less than 2% of peaks lacked either a PD or HD binding site, and these enhancers contained AP-2, KLF/SP1, and/or SOX binding site motifs. Genes associated with PAX3 peaks were commonly associated with pathways involved in transcription, RNA modification, and cell growth. While the majority of PAX3 peaks were within 5 Kb of the start sites of genes, 13.1% were over 50 Kb away from connected genes. These genes were commonly found in pathways involved in neuronal growth, function, and signaling. In this first report to employ a non-biased screen of PAX3 regulated genes in melanoma cells, there were several similarities and differences from prior gene candidate approaches. This highlighted shared cellular pathways as well as the heterogenetic variation of melanoma cells between donors. Here, we revealed several previously unknown potential gene targets of PAX3 and provide some insight into the wide functions of PAX3 in the regulation of melanoma growth, migration, and survival.

## 2. Materials and Methods

### 2.1. Cell Culture and Harvest

Human melanoma cell line SK-MEL-5 was obtained by the National Cancer Institute (NCI) Cell repository [RRID: CVCL_0527]. Cells were cultured in DMEM (Gibco, Waltham, MA, USA) with addition of 10% FBS (Corning/Mediatech, Inc. Woodland, CA, USA) without antibiotics. Histological analysis by DAPI staining of SK-MEL-5 cells was used to verify lack of mycoplasma contamination.

### 2.2. Cleavage Under Targets and Release Using Nuclease (CUT&RUN)

CUT&RUN [45,46] was performed using the CUTANA CUT&RUN kit (Epicypher, Durham, NC, USA, Cat.# 14-1048) following the manufacturer’s instructions with minor modifications. Briefly, 5 × 10^5^ cells were added to 10 μL activated magnetic Concanavalin A-coated beads per reaction. After a 10 min incubation, the supernatant was removed and cell–bead complexes were resuspended in cold buffer containing 0.02% Digitonin. Appropriate antibodies (Table 1) were added to reactions and incubated overnight at 4 °C with mixing. After washing beads twice, 2.5 μL pAG-MNase was added per reaction and incubated. pAG-MNase was activated by addition of 1 μL of 100 mM calcium chloride to 50 μL fresh buffer per reaction and incubated at 4 °C for 2 h with mixing followed by addition of 33 μL Stop Buffer and further incubation at 37 °C for 10 min. Released DNA fragments were treated with 0.2% SDS and 0.5 ug Proteinase K, incubated at 50 °C for 1 h, then purified by phenol/chloroform-extraction and ethanol precipitation. Fragment sizes were analyzed using a 2100 Bioanalyzer (Aligent, Santa Clara, CA, USA).

### 2.3. CUT&RUN Library Preparation and Data Analysis

All CUT&RUN libraries were generated using the NEBNext Ultra II DNA Library Prep Kit for Illumina (New England Biolabs (NEB), Ipswich, MA, USA, Cat.# E7645S) with NEBNext Multiplex Oligos for Illumina (NEB Cat.# E6440S). Libraries for histone CUT&RUN assays were performed per manufacturer’s instructions, except the NEBNext End Prep step temperature was reduced to 50 °C. Libraries for PAX3 CUT&RUN were performed as described [47]. Post library QC was achieved by fragment analysis on 2100 Bioanalyzer (Aligent). Libraries were pooled at equimolar concentrations and sequenced with 75 bp paired-end reads on an Illumina NextSeq550 platform (Illumina, Inc., San Diego, CA, USA). Rabbit anti-IgG control was used to account for non-specific binding and background signal. Reads were trimmed using Cutadapt (v4.8) and QC of trimmed files was implemented using MultiQC (v1.11) [48,49]. Trimmed reads were aligned against the hg19 genome assembly using Bowtie2 (v 2.4.5) [50]. Read-alignment BAM files were filtered using SAMtools (v1.8) [51] and PCR duplicates removed using Picard (v3.1.1) before BAM to BED and BigWig conversions were performed (BEDTools, v2.31.1) [52]. Peak calling was performed on BED files using MACS2 (v2.2.7.1) narrow peaks for PAX3 analysis and broad peaks for histone analyses [53]. The visualization of CUT&RUN tracks was generated using IGV (v2.15.4) [54].

### 2.4. ATAC-Seq Processing

Raw ATAC-seq data from the SK-MEL-5 cell line was downloaded from the GEO database (accession SRX13052791) and processed using a custom Snakemake (v7.32.4) pipeline [55,56,57]. The ATAC-seq had been performed in duplicate on 55,000 SK-MEL-5 cells using standard methodology and sequenced on an Illumina HighSeq 4000 instrument [56]. Reads were trimmed using Trimmomatic (v0.39) and QC of trimmed reads was implemented with FastQC (v0.12.1) [58,59]. Trimmed reads were aligned to the hg19 genome using Bowtie2 (v2.5.3) and mitochondrial reads were filtered out with SAMtools (v1.19.2). To account for Tn5 transposase binding characteristics alignments were shifted +4 bp and −5 bp on the forward and reverse strand, respectively using deepTools alignmentSieve (v3.5.4) [60]. Peaks were called using MACS2 (v2.7.1) with -nomodel. Identification of reproducible peaks was achieved by intersecting the two replicates with a 50% overlap requirement using BEDTools (v2.31.1) and filtering against the ENCODE blacklist regions.

### 2.5. Identification and Classification of PAX3 Binding Motifs

PAX3 peak coordinates were obtained from MACS2 narrow peak files generated from the CUT&RUN analysis and annotated with ChIPseeker (v1.38.) [61]. Peaks were extended by ±150 bp to capture the full mono-nucleosome using GenomicRanges (v1.54.1) [62]. The corresponding nucleotide sequences were extracted using the getSeq function from BSgenome.Hsapiens.UCSC.hg19 (v1.4.3) using R (v4.3.0). Blacklist regions (ENCODE hg19 v2) were filtered using rtracklayer (v1.62.0) to exclude artifactual or repetitive sequences [63,64]. Since PAX3 is known to bind via PD or HD, the processed PAX3 peak nucleotide files were analyzed for those motifs using regular expression-based screening. Nucleotide sequences were scanned for PD motifs (T[T/C][C/A][C/T][G/C][G/C], GTCA[C/T]GG) and HD motifs (TAAT[N_2,3_]ATTA and minor variants) using Biostrings (v2.7.0) [65]. Results of motif scanning were classified hierarchically as PD, HD, PDHD (possessing both PD and HD), or None.

### 2.6. Gene State Assessment and Classification

Histone Post-Translational Modification (PTM), and ATAC-seq binding sites were annotated with ChIPseeker (v1.38.). TSS were defined as regions spanning 2 Kb upstream and downstream of the annotated gene start sites, while gene regions were defined by extending gene body coordinates by 5 Kb on each side to capture proximal regulatory elements, except for H3K27me3. Using an in-house R script, each gene associated with a PAX3 peak was evaluated and classified hierarchically based upon associated histone PTMs and chromatin accessibility (Table 2).

### 2.7. Data Processing and Analysis

A small number of PAX3 annotated genes (22 of 837 genes or 2.6%) were found to not be readily assignable to any of the five gene state groupings. These were manually cross-checked and re-classified as appropriate. Gene Ontology (GO) enrichment analysis of Biological Processes (BP) and Molecular Function (MF) were performed using Metascape [66] and clusterProfiler [67] using the Benjamini–Hochberg adjusted p-values to determine the top 10 enriched pathways. All plots were generated with ggplot2 (v3.5.2) using R (v4.4.2) [68].

## 3. Results

### 3.1. PAX3-Bound Cistromic Regions in Melanoma Cells

**Figure 1:**
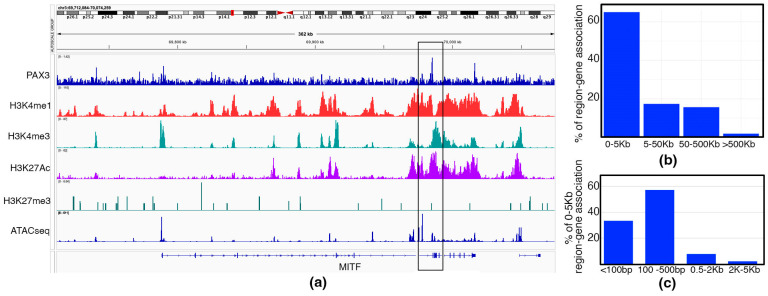
Analysis of SK-MEL-5 cell cistromic signatures. (**a**) Example of CUT&RUN and ATAC-seq data at the *MITF* locus, MACS2 peak (boxed), with antibodies against PAX3, H3K4me1 (Active enhancer), H3K4me3 (Active promoter), H3K27Ac (Active chromatin), H3K27me3 (Repressed chromatin).; (**b**,**c**) distance of PAX3 peaks from TSS, shown as the percentage of total peaks (**b**) and the total percentage of peaks 0–5 Kb from the TSS (**c**).

What genes and enhancer elements are controlled by PAX3 in melanoma is not well understood. To address this, we performed CUT&RUN to reveal the cistrome of PAX3 in SK-MEL-5 melanoma cells. First, two PAX3 antibodies, one monoclonal [Developmental Studies Hybridoma Bank/University of Iowa, Iowa City, IA, USA, Cat. # Pax3] and one polyclonal [Invitrogen/ThermoFisher, Waltham, MA, USA, Cat. # 38-1801], were tested and validated by assessing the constructed libraries using a 2100 Bioanalyzer (Aligent) to determine effectiveness for use in CUT&RUN. Analysis showed that only the polyclonal PAX3 antibody was suitable for CUT&RUN. CUT&RUN was also performed using antibodies for H3K4me1 and H3K4me3 (Active or Poised enhancers and promoters), and H3K27Ac and H327me3 (Active or Repressed chromatin). To mark open chromatin, ATAC-seq tracks were added from previously published datasets for SK-MEL-5 cells [56]. Three rounds of CUT&RUN were performed on SK-MEL-5 cells. One sample that did not pass QC was excluded from the downstream analysis. A total of 837 PAX3 peaks were detected from two remaining replicates. (Supplementary Table S1). Several of these peaks linked to previously identified enhancer elements regulated by PAX3, including MITF (Figure 1a). The PAX3 peak in the MITF loci was located proximal to the promoter driving the melanocyte specific m-MITF isoform, with H3K4me1, H3K4me3, H3K27Ac, and ATAC-seq peaks and a lack of H3K27me3 peak. Annotation of the dataset revealed that the vast majority of PAX3 peaks (more than 60%) were less than 5 Kb (<Kb) away from the TSS of the nearest annotated gene (Figure 1b). Over 90% of the peaks that were less than 5 Kb were within 0.5 Kb or less from the TSS (Figure 1c). This analysis uncovered a clear cistromic map of PAX3 regulation in SK-MEL-5 melanoma cells.

### 3.2. Binning of PAX3 Peaks with Paired and/or Homeodomain Binding Sites and with Histone Signatures

**Figure 2:**
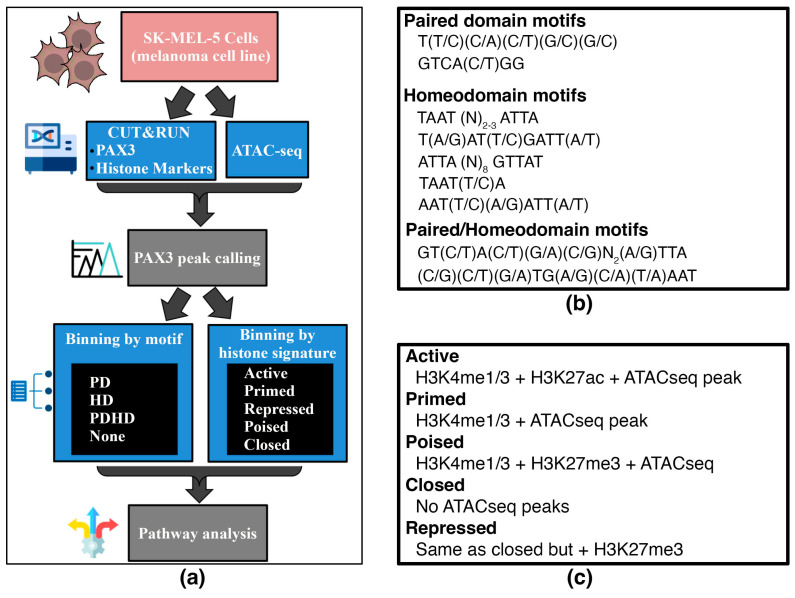
Schematic and criteria for the binning of PAX3 peaks with Paired and/or Homeodomain binding sites with histone signatures. (**a**) Schematic of workflow for characterizing peaks by motif and histone PTM signatures; (**b**) sequences of PD and HD motifs; (**c**) criteria for histone PTM marks for peaks. This binning is shown in tabular form in Table 2.

For this analysis, an in-house bioinformatic approach to examine PAX3 regulatory enhancers in terms of PAX3 binding motifs and histone signatures was employed (Figure 2a). First, the PAX3 peaks were identified, and then paired with the presence or absence of a PAX3-binding site within the peak (binning by motif), or with histone PTM tags and ATAC-seq peaks (binning by histone signature). For binning by motif, PAX3 has two DNA binding domains, PD and HD. How PAX3 interacts with DNA, and what cofactors cooperate on enhancers, differs between the PD and HD [1]. PAX3 peaks were sorted using custom R scripts containing PAX3 binding sites based on previously identified PD, HD, and dual PDHD sites [34,69,70,71,72,73,74,75,76,77] or peaks containing none of these sequences (Figure 2b). The PD binding site is difficult to predict due to the wobbly binding preferences of this domain, but has been determined through binding site selection assays, electrophoretic mobility shift assays, and DNA binding interference experiments [34,70,71,73,78]. The major HD site found in PAX family proteins is TAAT (N)_2–3_ ATTA, but there are several minor sites identified (referred to as HD_minor_, HD half-sites, H.12) identified for PAX3 and other PAX proteins through in vitro assays and crystal structure analysis [75,76]. While the PD and HD often bind independently, there is also evidence of crosstalk during DNA binding of either domain, as well as enhancer elements with dual PDHD sites. While many of the PDHD sites identified show a high binding affinity by the translocation product PAX3-FOXO1 or paralog PAX7, there is evidence that PAX3 can interact with PDHD sites as well, including the HP.E_0_ pioneer site [72,74,77]. For binning by histone signature, gene and promoter regions proximal to PAX3 peaks were classified as Active, Poised, Primed, Repressed, or Closed based on histone marks and accessibility (ATAC-seq) using previously identified signatures (Figure 2c) [79,80,81,82].

### 3.3. PAX3 Commonly Binds to Enhancers of Active Genes Through the Paired Domain in SK-MEL-5 Cells

**Figure 3:**
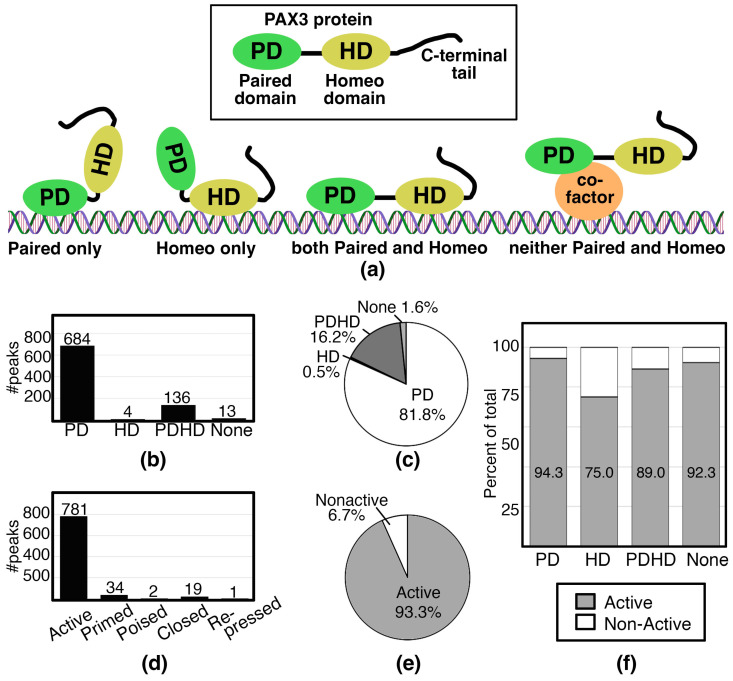
PAX3 binds to DNA primarily through the Paired Domain on enhancers affiliated with active genes. (**a**) Schematic representing the definition of PD, HD, PDHD, and None peaks; (**b**,**c**) number (**b**) or percentage (**c**) of peaks containing motifs for PD, HD, or PDHD, or none of these sites.; (**d**,**e**) number (**d**) or percentage (**e**) of peaks identified as Active, Primed, Poised, Closed, or Repressed chromatin; (**f**) percent of each peak identified as PD, HD, PDHD, or None that have Active (grey) or Non-Active (white) chromatin.

**Figure 4:**
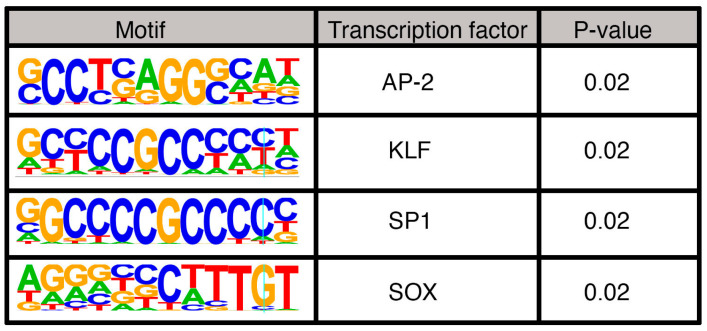
Motifs identified in PAX3 peaks without PD, HD, or PDHD sites.

After analyzing the PAX3 peaks using the criteria outlined in Figure 2, the peaks were sorted based on motif and histone signature. First, peaks were analyzed for PD, HD, and PDHD sites (Figure 3a). In SK-MEL-5 cells, the peaks predominantly contained PD sites, with 684 out of 837 peaks (81.8%) with this motif (Figure 3b,c). This included a previously identified PAX3 site in a RET gene enhancer [28]. Peaks containing a HD site (either HD only, coupled with a PD site, or a combined PDHD motif) were less common, comprising 16.7% of the peaks (Figure 3b,c). These peaks included a previously identified PAX3 site in a MITF gene enhancer [83,84]. A minority of the peaks (13 peaks, 1.6%) did not have a PD nor a HD binding motif (“None” peaks) (Figure 3b,c) and it is assumed that PAX3 is interacting with a cofactor that is recruiting PAX3 to the DNA (Figure 3a). To identify potential binding partners, HOMER motif analysis was performed on these 13 peak sequences. The None peaks contained AP-2, KLF, SP1, and/or SOX motifs (Figure 4).

In terms of histone signature, the majority of PAX3 peaks in the SK-MEL-5 cells have an Active signature. The PAX3 peaks were identified to be Active for 781 peaks, or 93.3% of total PAX3 peaks (Figure 3d,e). Unsurprisingly, sorting motifs (PD, HD, PDHD, None) by histone markers yielded mostly Active histone marks across all motif groups, due to the high number of peaks with Active histone marks (Figure 3f). While only 6.7% of the peaks were classified as Non-Active (identified as a Primed, Poised, Closed, or Repressed gene), motifs not classified as PD only (HD, PDHD, None) were overrepresented in this group, comprising 30.4% of the Non-Active genes (17 of 56 total peaks). Overall, our findings are that PAX3 is interacting with active gene enhancers directly through the Paired DNA binding domain.

### 3.4. Pathway Analysis of Potential PAX3 Downstream Genes in SK-MEL-5 Melanoma Cells

**Figure 5:**
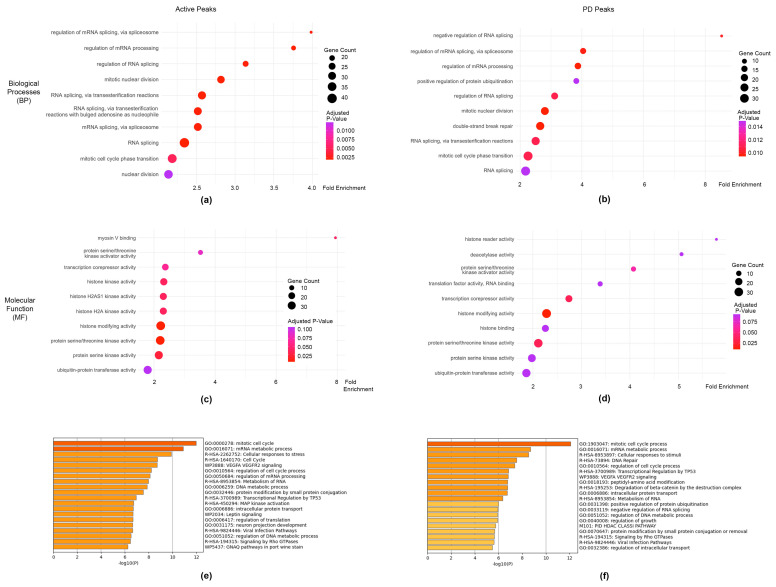
Pathway analysis of genes associated with PAX3 cistromic peaks. (**a**,**b**) Top 10 Gene Ontology (GO) pathways in Biological Process (BP) pathways for Active (**a**) and PD (**b**) groups; (**c**,**d**) GO pathways for Molecular Function (MF) for Active (**c**) and PD (**d**) groups.; (**e**,**f**) top 20 ontological terms from Metascape [66].

Comparative GO enrichment analysis by clusterProfiler found little difference between the most significant Biological Process (BP) and Molecular Function (MF) pathways in PAX3 genes from the Active and PD classification groups, with 70% of the top 10 BP pathways and 60% of the top 10 MF pathways shared between both groups (Figure 5a–d, Supplementary Tables S2 and S3). The GO:BP analysis revealed a substantial overlap between the two gene sets for RNA processing and cell cycle regulation, suggesting a shared functional signature of transcriptional and proliferative activity. Furthermore, GO:MF analysis showed considerable overlap in protein kinase activity, transcriptional corepression, histone modification, and ubiquitin-mediated processes, indicating commonality between the two gene sets in gene expression regulation and protein turnover. Presumably these results are due to both Active and PD group classifications containing the majority of the PAX3 genes which would result in a high degree of overlap between those groups. GO analysis of the PDHD + HD gene set found that no pathways were significantly enriched, likely due to the smaller number of genes present in that gene set (Supplementary Table S4).

Further analysis of the Active and PD pathways and processes was conducted in Metascape, which provides enrichment analysis using additional ontology terms including from the Broad Institute’s MSigDB [66]. Parallel with the clusterProfiler results, there was a substantial overlap in RNA processing and cell cycle regulation pathways with the Active and PD gene sets sharing 50% of the top 20 significant pathways between them (Figure 5e,f). Overall, the genes associated with PAX3 peaks fell into several pathways involved with transcription, RNA modification, and cell growth.

### 3.5. Genes Associated with Distal PAX3 Enhancers Have a Neuronal Signature

**Figure 6:**
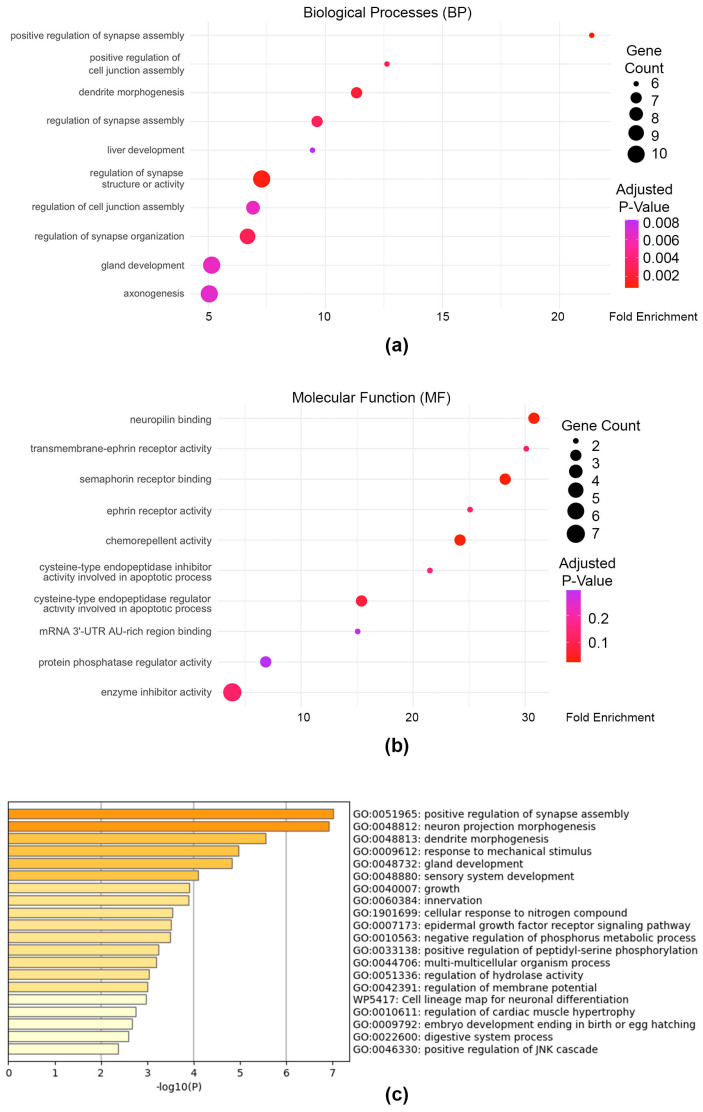
Pathway analysis of genes associated with PAX3 enhancers that are over 50 Kb from transcriptional start sites. (**a**) Gene Ontology (GO) pathways in Biological Process (BP) pathways of Distant genes, or genes with PAX3 peaks over 50 Kb from Transcriptional Start Sites; (**b**) GO pathways for Molecular Function (MF) for Distant gene set; (**c**) top 20 ontological terms from Metascape [66].

Since 13.1% of the PAX3 peaks were found to be ≥50 Kb from the TSS of the genes they annotated to, we performed separate GO-based pathway enrichment analyses to determine the pathways they influenced (Figure 6, Supplementary Table S5 and S6). Interestingly clusterProfiler GO:BP analysis found that the Distant gene set comprising distal PAX3 enhancers was significantly overrepresented in biological processes associated with neuronal development, cell adhesion, junction regulation, and developmental processes (Figure 6a,b). Meanwhile GO:MF analysis revealed enrichment for several signaling and regulatory activities such as neuropilin and ephrin receptor binding and semaphorin receptor activity. Metascape-based BP pathway enrichment analysis also found significant gene set overrepresentation in neuronal development, synaptic organization, and tissue morphogenesis pathways, which was a large departure from the Active and PD results (Figure 6c). These results suggest that genes with distal PAX3 enhancers ≥50 Kb from TSS coordinate neuronal differentiation, tissue development, and adaptive signaling processes, and may reflect a reactivation of neural crest-derived programs.

## 4. Discussion

This report is the first to investigate PAX3 genomic interaction in a melanoma cell line. We focused on SK-MEL-5 cells since there are already databases available containing cistromic information, and that this cell line has the common BRAF valine 600 to glutamic acid mutational change coupled with a wild-type N-RAS gene [43,44]. In these cells, we identified several known melanoma PAX3 targets, including *MITF* and *BRN2/POU3F2* [5,8,85]. However, several other downstream targets were absent from our dataset. These include validated melanoma targets, such as *CXCR4*, *MET* and *TYRP1* [9,11,26,36]. Conversely, some of the known PAX3 target genes that had PAX3 peaks in SK-MEL-5 cells were also variably controlled by PAX3 in other cell lines. An example of this is *MITF*, which is not always regulated by PAX3 in melanoma [4,11,86]. This is likely due to the heterogenicity of melanoma cells, and that these prior reports did not test SK-MEL-5 cells. Further, while we chose SK-MEL-5 cells as a representative of melanoma cells due to their BRAF mutation and cellular morphology, a difference between this cell line and other established lines is that the donor was significantly younger (24 years old) and has a CDKN2A mutation [87]. The role of PAX3 may differ depending on how melanoma is induced and promoted, such as genetic, environmental or other variables.

Most of the peaks contain PD, while only a small minority have a HD (140 peaks), and even less with HD only (4 peaks) (Figure 3b,c). This was not surprising, since it was previously described that PAX3 was not effective in inducing transcription from enhancer elements with only HD motifs in myogenic cells [14]. Further, while the majority of the genes associated with PAX3 peaks in this study are Active, there are a minority of genes that are Non-Active, and HD containing sites are overrepresented in this peak set (Figure 3). There is a precedent for Paired-type HD to act as repressors, often working with TLE/Groucho factors [88]. PAX3 functions as a transcriptional repressor for some genes such as *MBP* and *Dct* [29,89]. PAX3 interacts with TLE4 in murine melanocyte stem cells, but it is not clear through which domain PAX3 is interacting with the enhancers of either *MDP* or *Dct* [29,89]. We do not see evidence of direct gene repression in this study. It may be that in this cell line gene repression is indirect, since several genes were in pathways involved in transcriptional repression (Figure 5b,e).

There was a small minority of peaks identified that lacked either HD or PD sites. Motif analysis of these peaks revealed AP-2, KLF/SP1, and SOX sites (Figure 4). SOX family members are common PAX protein co-factors; in melanocytes and melanoma, PAX3 interacts with SOX10 and drives a number of downstream genes, such as MITF, MET, and RET [1,11,28,83,90]. AP-2, also known as TFAP2 family members, are known to promote melanocyte development and maturation, as well as function in melanoma [91,92,93]. No prior connection is known between PAX proteins and TFAP2 factors, except for some evidence supporting an upstream role for promoting TFAP2 expression in *xenopus* [94]. PAX3 is known to drive expression of genes involved in both an invasive and proliferative phenotype in melanoma, and TFAP2A can work as a rheostat switch between phenotypic groups [95]. While an interaction between PAX3 and TFAP2 factors in driving phenotype switching is an interesting possibility, presently it is unknown if these proteins cooperate in this way. We also found KLF/SP1 sites. SP1-like proteins and Krüppel-like factors (KLF) are highly related zinc-finger transcription factors that bind to GC-rich promoter regions [96]. These factors are known to regulate several cellular functions, including cell growth, survival, and differentiation. While there is currently no known connection between PAX3 and KLF/SP1 factors, SP1 is known to compete with TFAP2 factors through transcriptional steric interference and act as a repression/activation switch [97]. It may be a clue into a potential mechanism of a PAX3/TFAP2/SP1 rheostat between cell phenotypes of functions.

The majority of documented PAX3-targeted enhancers are located in the 5′ proximal promoter, within the first intron, or in the 3′ UTR [5,10,26,33,36,37], which was supported by our CUT&RUN analysis where the majority of PAX3 peaks were near gene TSS in this cell line (Figure 1). One caveat is that the enhancer with the closest proximity to a gene does not definitively regulate that gene. Indeed, several known PAX3 enhancer sites in somites and muscle cells are more than 5 Kb away from their regulated gene’s TSS; for example *MYF5* (57.5 Kb and 111 Kb upstream from the start site) [98,99] and *DMRT2* (11 Kb upstream) [100]. While we predict regulated genes in this report based on proximity, each enhancer would need additional experimentation to validate their regulation by PAX3. Furthermore, nearly 40% of our enhancers were more than 5 Kb away from a TSS, with nearly 20% over 50 Kb away (Figure 1b). The analysis of genes associated with these distal enhancers revealed a neuronal pathway profile, with terms linked to neuronal growth, function, and signaling (Figure 6). This is a vastly different signature than for the other PAX3 enhancers, which fall into transcription, RNA modification, and cell growth pathways (Figure 5). The genes that are associated with these distal enhancers include ephrins, semaphorins, and SLIT/TRK proteins. Several of these proteins are involved in neural crest differentiation, which requires PAX3 function for normal development and differentiation. This raises a possibility that PAX3 regulated enhancers involved with differentiation are located distally from genes in neural crest populations, in parallel to what is seen in somite and muscle precursors.

## 5. Conclusions

In SK-MEL-5 melanoma cells, PAX3 is bound to DNA elements associated with genes involved with transcription, RNA modification, cell growth, and neuronal growth, function, and signaling. This is the first study to perform a non-biased genomic screen to reveal a PAX3 cistromic signature in melanoma cells.

## Figures and Tables

**Table 1:** Antibodies used for CUT&RUN assay. All antibodies were rabbit raised antibodies against the indicated targets.

Antibody	Supplier	Catalog Number
PAX3	Invitrogen	38-1801
IgG Control	EpiCypher	13-0042k
H3K4me3	EpiCypher	13-0041
K3K4me1	EpiCypher	13-0057
H3K27me3	EpiCypher	13-0055
H3K27ac	Millipore	MABE647

**Table 2:** Classification of gene state based upon chromatin marks and accessibility. Note, for Active, Primed, and Poised classifications, the presence of either H3K4me1 or H3K4me3, or both together, are required to satisfy the classification criteria. + = presence, − = absence, +/− can be either present or absent.

Classification	H3K4me1	H3K4me3	H3k27me3	H3K27ac	ATAC Peak
Active	+	+	−	+	+
Primed	+	+	−	−	+
Poised	+	+	+	−	+
Repressed	−	−	+	−	−
Closed	+/−	+/−	−	+/−	−

## Data Availability

The original data presented in the study are openly available in NCBI Sequence Read Archive at Accession number PRJNA1260960, https://www.ncbi.nlm.nih.gov/bioproject/PRJNA1260960 accessed on 9 May 2025.

## Supplementary Materials

The following supporting information can be downloaded at: https://www.mdpi.com/article/10.3390/genes16050577/s1, Supplementary Table S1. Pax3 peaks categorized to histone marks and binding sites; Supplementary Table S2. Active GO, BP and MF merged (clusterProfiler); Supplementary Table S3. PD GO, BP and MF merged (clusterProfiler); Supplementary Table S4. PDHD_HD GO, BP and MF merged (clusterProfiler); Supplementary Table S5. Genes with PAX3 peaks greater than 50Kb from TSS (distant); Supplementary Table S6. Distant GO, BP & MF merged (clusterProfiler).

## Abbreviations

The following abbreviations are used in this manuscript:

BP	Biological Processes
CNS	Central Nervous System
CUT&RUN	Cleavage Under Targets & Release Using Nuclease
FP-RMS	Fusion Positive Rhabdomyosarcoma
GO	Gene Ontology
HD	Homeodomain
MF	Molecular Function
NCI	National Cancer Institute
PD	Paired Domain
PDHD	Homeodomain and Paired Domain
PTM	Post-Translational Modification
TSS	Transcriptional Start Site
